# Trends in the complexity of self-harm hospitalisations over 11 years at a major regional hospital in Victoria, Australia: a retrospective study

**DOI:** 10.3389/fpsyt.2025.1573824

**Published:** 2025-04-30

**Authors:** George Mnatzaganian, Rebecca Giallo, Reagan Martin, Angela Crombie, Ziad Nehme, Belinda Delardes, David Burns, Susan Furness, Tim Lenten, Renee Sharples, Lisa Hanson, Rachel Huxley

**Affiliations:** ^1^ La Trobe Rural Health School, La Trobe University, Bendigo, VIC, Australia; ^2^ School of Psychology, Faculty of Health, Deakin University, Melbourne, VIC, Australia; ^3^ Research & Innovation, Bendigo Health, Bendigo, VIC, Australia; ^4^ Centre for Research and Evaluation, Ambulance Victoria, Melbourne, VIC, Australia; ^5^ School of Public Health and Preventive Medicine, Monash University, Melbourne, VIC, Australia; ^6^ Clinical Learning and Development, Bendigo Health, Bendigo, VIC, Australia; ^7^ Nursing Mental Health Services, Bendigo Health, Bendigo, VIC, Australia; ^8^ Faculty of Health, Deakin University, Melbourne, VIC, Australia; ^9^ The George Institute for Global Health, University of New South Wales, Sydney, NSW, Australia

**Keywords:** ambulance, AR-DRG, hospitalisation, self-harm, severity of illness, sex, suicide, trends

## Abstract

**Background:**

This population-based study explored the Australian Refined Diagnosis Related Groups (AR-DRG) complexity of self-harm admissions over time at a major regional hospital in Victoria, Australia. It also assessed the prehospital paramedic management of such admitted patients.

**Methods:**

Self-harm admissions at the hospital from January 1, 2010, to December 31, 2020, were included, excluding accidental injuries and children under 10. Hospital records were linked with Ambulance Victoria electronic patient care records. Trends in the age- and social status-adjusted AR-DRG complexity of self-harm hospitalisations by sex were analysed using Dickey-Fuller and MacKinnon tests. Odds of presenting with a major complexity were modelled using the Generalised Estimating Equations approach.

**Findings:**

Overall, 2,000 individuals (58.6% female, mean age at last admission 36.9 ± 18.6 years), contributing to 2,808 admissions, were included in the study. The proportion of self-harm admissions with a major AR-DRG complexity significantly increased over time, from 9.3% in 2010 to 43.5% in 2020 (p<0.001). Both increased complexity and intensive care unit admissions were observed in both males and females. The use of multiple self-harm methods also rose over time. Of the 2,000 patients, 1,416 (70.8%) sought emergency services assistance within 30 days of hospital admission, with 139 (9.8%) not transported to the emergency department. These non-transported patients had higher odds of presenting to the hospital within one month of the prehospital paramedic assessment with more complex conditions (odds ratio 2.04, 95% confidence interval 1.39-2.99, p<0.001) and longer hospital stays compared to those who were transported.

**Interpretation:**

Our findings indicate a trend toward more severe cases of self-harm over time, observed in both males and females, with an increase in the use of multiple self-harm methods. Additionally, our results suggest that paramedic non-transports to the emergency department should be re-evaluated, as these patients experience worse outcomes.

## Introduction

Each year, millions of people worldwide engage in self-harm, with some attempting suicide, though many of these do not die from their injuries (1). Self-harm and suicide affect all individuals across all demographics, regardless of sex, gender, age, ethnicity, religion, or country of birth. However, trends show that different groups experience and respond to self-harm differently. Studies suggest that women are more likely to engage in non-suicidal self-injury (NSSI) (2), while men have a higher likelihood of dying by suicide (3). Recent data from the Australian Institute of Health and Welfare (AIHW) show that in the 2022–23 period, females had higher hospitalisation rates for intentional self-harm than males (4). Self-harm admission rates also vary by age, with younger Australians experiencing higher prevalence. In 2022-23, the rate for females aged 15–19 years was 499 per 100,000, compared to 127 per 100,000 for males in the same age group. Similarly, for females aged 20–24 years, the rate was 289 per 100,000, while males in this age group had a rate of 122 per 100,000 (4). Approximately 16.7% of Australians aged 16–85 have experienced thoughts of ending their lives at some point, with around 7.4% having made a suicide plan and 4.9% attempting suicide during their lifetime (5). Differences in Australian self-harm hospital admissions over time also vary by residential area with hospitalisations decreasing in major cities (111 to 107 per 100,000, in 2012-13 and 2020-21, respectively) but increasing in outer regional (136 to 149 per 100,000) and rural remote locations (146 to 171 per 100,000) (6).

While there is evidence of increased self-harm admissions, it is unclear whether these admissions have become more complex over time. In the context of hospital care, self-harm cases are categorised into Diagnosis-Related Groups (DRGs), which reflect the complexity and resource utilisation involved in patient care and are used for hospital service reimbursement (7). The complexity level of a presentation is determined by factors such as illness severity, comorbidities, anticipated procedural needs, resource utilisation (e.g., medical tests, nursing care), and expected length of stay. Patients classified under high complexity often require more intensive care and greater resources (7, 8). However, there is limited research exploring how the complexity of DRGs in self-harm admissions has changed over time, particularly in regional or non-metropolitan settings. Existing literature tends to focus on the clinical aspects of self-harm or general trends in hospital admissions, without specifically addressing shifts in DRG complexity related to self-harm cases (9, 10). Understanding these changes is critical for improving resource allocation, healthcare planning, and patient care strategies. This study aimed to address this gap by examining trends in DRG complexity for self-harm admissions at a major regional hospital in Victoria, Australia, over 11 years. Paramedic management in the prehospital setting and patient outcomes were also analysed.

## Materials and methods

### Study population and design

This population-based retrospective study included all individuals aged 10 years and older who were hospitalised at a large regional health service provider in Victoria, Australia, following intentional or undetermined self-harm between January 2010 and December 2020. Cases were identified using the International Classification of Diseases, 10^th^ Revision, (Australian Modification) (ICD-10-AM) codes of X60 – X84 and Y10 – Y34. Presentations due to accidental poisoning or injury were excluded. Similarly, individuals with missing information on age, sex, or methods of self-harm were excluded.

The health service provider has a catchment area of 58,957 km^2^, covering 25.9% of the land mass of Victoria. It includes 10 local government areas and serves an estimated population of 315,241 individuals (as of 2021).

### Study variables

Hospital patient records were linked with prehospital electronic Patient Care Records (ePCR) from Ambulance Victoria, the primary provider of emergency medical services in the state of Victoria. The linkage was deterministic, using the patient’s full name and date of birth, and was performed by the data custodians without revealing any personal information to the investigating researchers. In this study, all ambulance attendances within 30 days prior to hospital admission for self-harm were included, as an *a priori* decision was made to capture ambulance attendances that did not result in transport to the hospital. The collected hospital data included information on admission characteristics, the patient’s residential postcode, Australian Refined Diagnosis-Related Groups complexity classification (7), self-harm method used, admission to an intensive care unit (ICU) or coronary care unit (CCU), length of hospital stay, and in-hospital mortality.

The Independent Hospital Pricing Authority (IHPA) is tasked with creating AR-DRG classification systems that offer a consistent, nationwide approach for categorising patients, their treatments, and the associated costs (7). The AR-DRG classification system provides a clinically relevant method for grouping patients based on the number and types of treatments received during acute care episodes, and the resources needed for these treatments. AR-DRGs use diagnosis and intervention codes, along with other routinely collected data, to classify episodes of care for admitted patients. The AR-DRG classifications varied over time: Version 6.0 for 2010, Version 6.0x for 2011-2012, Version 7.0 for 2013-2015, Version 8.0 for 2016-2017, Version 9.0 for 2018-2019, and Version 10.0 for 2020 (8).

Socioeconomic status was based on the 2021 Census-derived residential postcode-based Socio-Economic Indexes for Areas – Index of Relative Socio-Economic Disadvantage (SEIFA-IRSD), which is a composite score that ranks geographic areas across Australia according to their relative socioeconomic disadvantage, with higher scores indicating less socioeconomic disadvantage (11). Geographical remoteness classification (metropolitan, regional, rural, and remote areas) was based on the Modified Monash Model (MMM) (12).

### Statistical analyses

The method used and characteristics of patients admitted for self-harm were analysed by sex and repeat admission over time. A Chi-square test compared categorical variables, while a Kruskal-Wallis test compared mean ranks.

For each year, the AR-DRG complexity of self-harm admissions was estimated, adjusting for age at admission and socio-economic disadvantage. Risk-adjusted trends in AR-DRG complexity over time by sex were tested using the Dickey-Fuller and MacKinnon tests (13, 14). To conclude that the time series was non-stationary, the MacKinnon approximate p value test needed to be insignificant (i.e., >0.05) (14). Trends in ICU or CCU admissions were similarly assessed.

Using unique admission episodes, the odds of having a major complexity upon admission for self-harm were analysed using the Generalised Estimating Equations (GEE) approach. An exchangeable covariance matrix was used to account for correlation and dependence between repeat admissions on the same individual over time while adjusting for age at admission, sex, geographical remoteness, year of admission, number of self-harm methods used, and management by paramedics in the prehospital setting.

To account for possible misclassification of the self-harm admission, we conducted a sensitivity analysis that excluded patients whose self-harm intent was unknown.

The analyses were performed using Stata/SE 18 (Stata Corp LP., College Station TX, USA).

## Results

**Figure 1:**
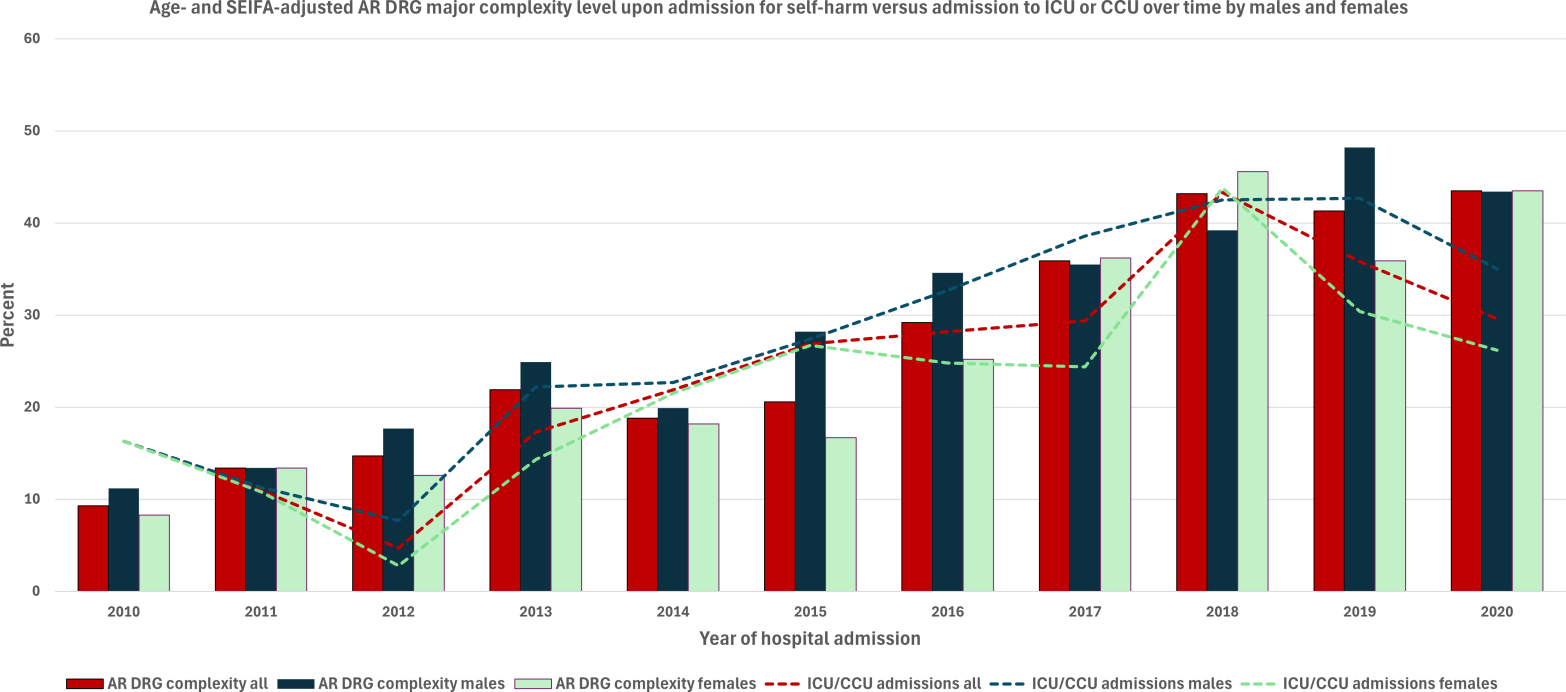
Age- and SEIFA-adjusted major AR-DRG complexity of self-harm admissions versus admission to an intensive or coronary care unit over time by sex.

Over the 11-year study period, three individuals were excluded for having other than male or female gender, leaving a total of 2,000 patients (58.6% female, mean age at last admission 36.9 ± 18.6 years) who were admitted for self-harm. Over 73.0% of the individuals belonged to the two lowest socioeconomic status quintiles, with this pattern consistent across both sexes. Females were more likely than males to be admitted via the emergency department (94.0% vs. 90.0%, p=0.006) and to have two or more repeat self-harm admissions over the 11-year study period (23.9% in females vs. 16.5% in males, p<0.001) (**Table 1**). Males, who were more likely than females to have their self-harm categorised as of unknown intention (28.2% vs. 16.1%, p<0.001), were also more likely to be admitted with major complexity (35.0% vs. 31.1%, p<0.001). Compared to males, females more commonly used poisoning from prescribed and over-the-counter medications, alcohol, gases, and chemicals for self-harm – a trend that was consistent across all age groups (**Table 2**). In contrast, males were more likely than females to use smoke, flames, hanging, strangulation, and sharp or blunt objects to self-harm. Although most males and females typically used a single method of self-harm, females were more likely to use multiple methods. In repeat incidents, both sexes significantly increased the use of multiple methods (**Table 3**). Over time, the age- and social disadvantage-adjusted AR-DRG complexity of self-harm admissions rose (MacKinnon approximate p-value for Z(t) = 0.5957), along with an increase in admissions to intensive care units or coronary care units for both sexes (**Figure 1**). The correlation (estimated using Spearman’s rho coefficient) between AR-DRG complexity and admission to ICU/CCU tripled over the years, rising from 0.151 before 2013 to 0.403 after 2017 (p<0.001).

**Table 1:** Characteristics of hospitalised individuals for self-harm by sex.

	All	Male	Female	P value
	N=2,000 (100.0%)	N=829 (41.4%)	N=1,171 (58.6%)	
Age (years) at last admission				<0.001
Mean (SD)	36.9 (18.6)	38.8 (17.9)	35.4 (18.9)	
Median (IQR)	33 (21, 50)	35 (23, 51)	31 (19, 49)	
Youngest, oldest	10, 96	10, 93	10, 96	
Age categories at last admission, n (%)				<0.001
<18	284 (14.2)	64 (7.7)	220 (18.8)	
18.0 – 24.9	413 (20.6)	164 (19.8)	249 (21.3)	
25.0 – 34.9	356 (17.8)	175 (21.1)	181 (15.5)	
35.0 – 44.9	311 (15.5)	137 (16.5)	174 (14.9)	
45.0 – 54.9	265 (13.2)	125 (15.1)	140 (12.0)	
≥55.0	371 (18.5)	164 (19.8)	207 (17.7)	
SEIFA-IRSD, n (%)				0.024
1st category - most disadvantaged	659 (32.9)	274 (33.1)	385 (32.9)	
2nd category	820 (41.0)	340 (41.0)	480 (41.0)	
3rd category	207 (10.3)	90 (10.9)	117 (10.0)	
4th category	267 (13.3)	96 (11.6)	171 (14.6)	
5th category – least disadvantaged	41 (2.1)	24 (2.9)	17 (1.4)	
Unknown	6 (0.3)	5 (0.6)	1 (0.1)	
MMM Remoteness classification, n (%)				0.009
Metropolitan areas	61 (3.0)	34 (4.1)	27 (2.3)	
Regional centres	1,112 (55.6)	440 (53.1)	672 (57.4)	
Rural or remote	821 (41.0)	350 (42.2)	471 (40.2)	
Unknown	6 (0.3)	5 (0.6)	1 (0.1)	
Number of unique self-harm admissions during 2010-2020, n (%)				<0.001
1 admission	1584 (79.2)	693 (83.6)	891 (76.1)	
2 admissions	260 (13.0)	90 (10.9)	170 (14.5)	
≥ 3 admissions	156 (7.8)	46 (5.6)	110 (9.4)	
Intention to self-harm, n (%)				<0.001
Intentional	1,578 (78.9)	595 (71.8)	983 (83.9)	
Intention unknown	422 (21.1)	234 (28.2)	188 (16.1)	
Highest listed AR-DRG complexity during 2010-2020, n (%)				<0.001
Minor / Intermediate	1,274 (63.7)	493 (59.5)	781 (66.7)	
Major	654 (32.7)	290 (35.0)	364 (31.1)	
Unknown	72 (3.6)	46 (5.6)	26 (2.2)	
Ever managed by a paramedic within 30 days before admission, n (%)	1,416 (70.8)	566 (68.3)	850 (72.6)	0.037

AR-DRG, Australian Refined Diagnosis Related Groups; IQR, interquartile range; MMM, Modified Monash Model; SD, standard deviation; SEIFA-IRSD, Socio-Economic Indexes for Areas – Index of Relative Socio-Economic Disadvantage.

**Table 2:** Methods ever used during 2010-2020 by sex and age category at last and most recent hospitalisation, percent.

	Males							Females						
	<18 N=64	18-24.9 N=164	25-34.9 N=175	35-44.9 N=137	45-54.9 N=125	>55 N=164	All ages N=829	<18 N=220	18-24.9 N=249	25-34.9 N=181	35-44.9 N=174	45-54.9 140	>55 N=207	All ages N=1171
Poisoning methods														
**Method 1**	37.5	43.3	48.0	52.6	52.8	40.8	**46.3**	38.6	58.6	65.7	69.0	67.1	50.2	**57.0**
**Method 2**	50.0	20.7	13.7	16.1	21.6	16.5	**20.0**	61.4	51.8	28.2	30.5	30.0	27.0	**39.8**
**Method 3**	18.7	11.6	20.0	13.1	15.2	27.4	**17.8**	18.2	20.1	12.7	18.4	20.0	25.6	**19.3**
**Method 4**	6.2	16.5	20.6	26.3	20.8	12.2	**18.0**	10.4	12.8	21.0	21.8	24.3	16.4	**17.0**
**Method 5**	3.1	7.9	13.7	24.1	19.2	14.6	**14.5**	6.4	12.4	18.8	24.7	19.3	15.0	**15.4**
**Method 6**	4.7	1.8	4.0	2.2	2.4	7.3	**3.7**	3.2	3.6	2.8	6.9	5.7	4.3	**4.3**
**Method 7**	3.1	1.8	2.3	0.7	1.6	2.4	**1.9**	1.8	2.8	3.3	5.2	5.0	2.4	**3.2**
Other than poisoning methods														
**Method 8**	21.9	15.8	22.3	19.0	16.8	21.3	**19.4**	10.9	9.6	13.8	8.1	7.9	11.1	**10.3**
**Method 9**	4.7	4.9	4.0	3.7	3.2	0.6	**3.4**	1.4	2.4	7.2	2.9	2.1	0.5	**2.7**
**Method 10**	1.6	3.1	8.0	5.8	7.2	7.3	**5.9**	0.5	2.0	2.8	0.6	1.4	6.8	**2.3**
**Method 11**	0.0	3.7	2.3	5.1	3.2	3.1	**3.1**	0.9	1.2	1.1	2.9	0.0	1.9	**1.4**
**Method 12**	1.6	2.4	5.1	1.5	3.2	5.5	**3.5**	1.4	4.8	5.0	1.1	3.6	3.9	**3.3**

Method 1: “Poisoning by and exposure to antiepileptic, sedative-hypnotic, antiparkinsonism and psychotropic drugs”.

Method 2: “Poisoning by and exposure to nonopioid analgesics, antipyretics and antirheumatics”.

Method 3: “Poisoning by and exposure to other and unspecified drugs, medicaments and biological substances”.

Method 4: “Poisoning by and exposure to narcotics and psychodysleptics”.

Method 5: “Poisoning by and exposure to alcohol”.

Method 6: “Poisoning by and exposure to other drugs acting on the autonomic nervous system”.

Method 7: “Poisoning by and exposure to other and unspecified chemicals and noxious substances”.

Method 8: “Self-harm by blunt/sharp object”.

Method 9: “Self-harm by hanging, strangulation and suffocation”.

Method 10: “Exposure to smoke, fire and flames or exposure to other gases and vapours”.

Method 11: "Self-harm by other specified means".

Method 12: “Self-harm by other unspecified means”.

Figures presented in bold font related to all ages in males and females.

**Table 3:** Characteristics and outcomes of self-harm admissions by sex and repeat admission.

Covariates	Male				Female			
	1 admission N=693 (83.6)	2 admissions N=90 (10.9)	≥3 admissions N=46 (5.6)	p	1 admission N=891 (76.1)	2 admissions N=170 (14.5)	≥3 admissions N=110 (9.4)	p
Age (years) at last admission				0.293				0.846
Mean (SD)	38.5 (18.2)	40.6 (16.9)	39.8 (16.2)		35.8 (19.2)	34.5 (18.3)	33.8 (16.7)	
Median (IQR)	35 (23, 51)	38 (30, 51)	38.5 (27, 53)		31 (19, 50)	28.5 (19, 47)	28 (21, 44)	
Youngest, oldest	10, 93	16, 88	13, 86		10, 95	13, 90	13, 96	
Intention to self-harm, n (%)				<0.001				<0.001
Intentional	470 (67.8)	79 (87.8)	46 (100.0)		711 (79.8)	164 (96.5)	108 (98.2)	
Intention unknown	223 (32.2)	11 (12.2)	0 (0.0)		180 (20.2)	6 (3.5)	2 (1.8)	
Highest AR DRG complexity during 2010-2020, n (%)				<0.001				<0.001
Minor / Intermediate	446 (64.4)	38 (42.2)	9 (19.6)		643 (72.2)	106 (62.3)	32 (29.1)	
Major	202 (29.1)	51 (56.7)	37 (80.4)		223 (25.0)	63 (37.1)	78 (70.9)	
Unknown	45 (6.5)	1 (1.1)	0 (0.0)		25 (2.8)	1 (0.6)	0 (0.0)	
Ever admission to ICU / CCU during 2010-2020, n (%)	179 (25.8)	51 (56.7)	37 (80.4)	<0.001	180 (20.2)	60 (35.3)	68 (61.8)	<0.001
Number of different methods ever used to self-harm, n (%)				<0.001				<0.001
1 method	498 (71.9)	35 (38.9)	7 (15.2)		584 (65.5)	50 (29.4)	12 (10.9)	
2 methods	130 (18.8)	27 (30.0)	12 (26.1)		203 (22.8)	61 (35.9)	37 (33.6)	
≥3 methods	65 (9.4)	28 (31.1)	27 (58.7)		104 (11.7)	59 (34.7)	61 (55.4)	
Poisoning method ever used to self-harm, n (%)	512 (73.9)	74 (82.2)	42 (91.3)	0.009	789 (88.5)	159 (93.5)	104 (94.5)	0.033
Other-than-poisoning method ever used to self-harm, n (%)	196 (28.3)	30 (33.3)	23 (50.0)	0.006	119 (13.4)	28 (16.5)	41 (37.3)	<0.001
Length of hospital stay, days				<0.001				<0.001
Mean (SD)	4.0 (11.9)	11.3 (17.6)	19.7 (21.8)		3.0 (8.4)	7.0 (20.1)	26.8 (56.0)	
Median (IQR)	1 (1, 2)	5 (2, 11)	13 (8, 22)		1 (1, 2)	2.5 (2, 5)	11 (5, 22)	
Shortest, longest	1, 202	1, 106	4, 126		1, 163	2, 244	3, 474	
Deaths during the index admissions, n (%)	6 (0.9)	2 (2.2)	0 (0.0)	0.271	15 (1.7)	5 (2.9)	1 (0.9)	0.470
In-hospital deaths (in this hospital) during 2010-2020, n (%)	62 (9.0)	9 (10.0)	4 (8.7)	0.907	55 (6.2)	13 (7.6)	5 (5.5)	0.592

AR-DRG, Australian Refined Diagnosis Related Groups; CCU, coronary care unit; ICU, intensive care unit; IQR, interquartile range; SD, standard deviation.

Of all individuals, 584 (29.2%) never requested paramedic assistance within 30 days of hospitalisation and either self-presented or were brought to the emergency department (ED) by others. Meanwhile, within the same 30-day period, 1,277 (63.8%) were assessed by paramedics in the prehospital setting and transported to the ED, while 139 (7.0%) were assessed but not transported. Individuals assessed by paramedics but not transported to the ED were significantly older than others in the sample, with a higher proportion of males and individuals living in rural or remote areas (**Table 4**). These 139 patients either self-presented to the ED or were brought by others within a few days of paramedic management in the prehospital setting, with a mean (standard deviation) of 2.4 (5.4) days, ranging from 0 (same day as paramedic assessment) to 24 days later. Upon ED presentation, 41.7% of these patients had an AR-DRG major complex condition. They also had longer hospital stays compared to individuals managed by paramedics in the prehospital setting and transported to the ED.

**Table 4:** Characteristics of self-harming individuals ever transported and not transported to the emergency department by paramedics’ assessment status.

	Self-presentations at ED without paramedics’ ever assessment N=584 (29.2)	Assessed by paramedics and transported to ED N=1,277 (63.8)	Assessed by paramedics but not transported to ED N=139 (7.0)	P value
Age (years) at last admission				<0.001
Mean (SD)	31.8 (17.4)	38.1 (18.4)	46.5 (19.5)	
Median (IQR)	26 (19, 41)	35 (22, 51)	45 (31, 61)	
Youngest, oldest	10, 95	11, 96	13, 92	
Age categories at last admission, n (%)				<0.001
<18	120 (20.6)	153 (12.0)	11 (7.9)	
18.0 – 24.9	155 (26.5)	247 (19.3)	11 (7.9)	
25.0 – 34.9	118 (20.2)	218 (17.1)	20 (14.4)	
35.0 – 44.9	69 (11.8)	216 (16.9)	26 (18.7)	
45.0 – 54.9	49 (8.4)	192 (16.0)	24 (17.3)	
≥55.0	73 (12.5)	251 (19.7)	47 (33.8)	
Sex, n (%)				0.045
Male	263 (45.0)	503 (39.4)	63 (45.3)	
Female	321 (55.0)	774 (60.6)	76 (54.7)	
MMM Remoteness classification, n (%)				<0.001
Metropolitan areas	16 (2.7)	40 (3.1)	5 (3.6)	
Regional centres	376 (64.4)	683 (53.5)	53 (38.1)	
Rural or remote	192 (32.9)	549 (43.0)	80 (57.6)	
Unknown	0 (0.0)	5 (0.4)	1 (0.7)	
Admission to ICU / CCU, n (%)	94 (16.1)	427 (33.4)	54 (38.8)	<0.001
AR-DRG major complexity, n (%)	116 (19.9)	364 (28.5)	58 (41.7)	<0.001
Total hospital length of stay, mean (SD)	4.9 (14.5)	5.6 (19.6)	10.2 (22.7)	<0.001
In-hospital death as recorded in participating hospital during 2010-2020, n (%)	30 (5.1)	102 (8.0)	16 (11.5)	0.015

**Figure 2:**
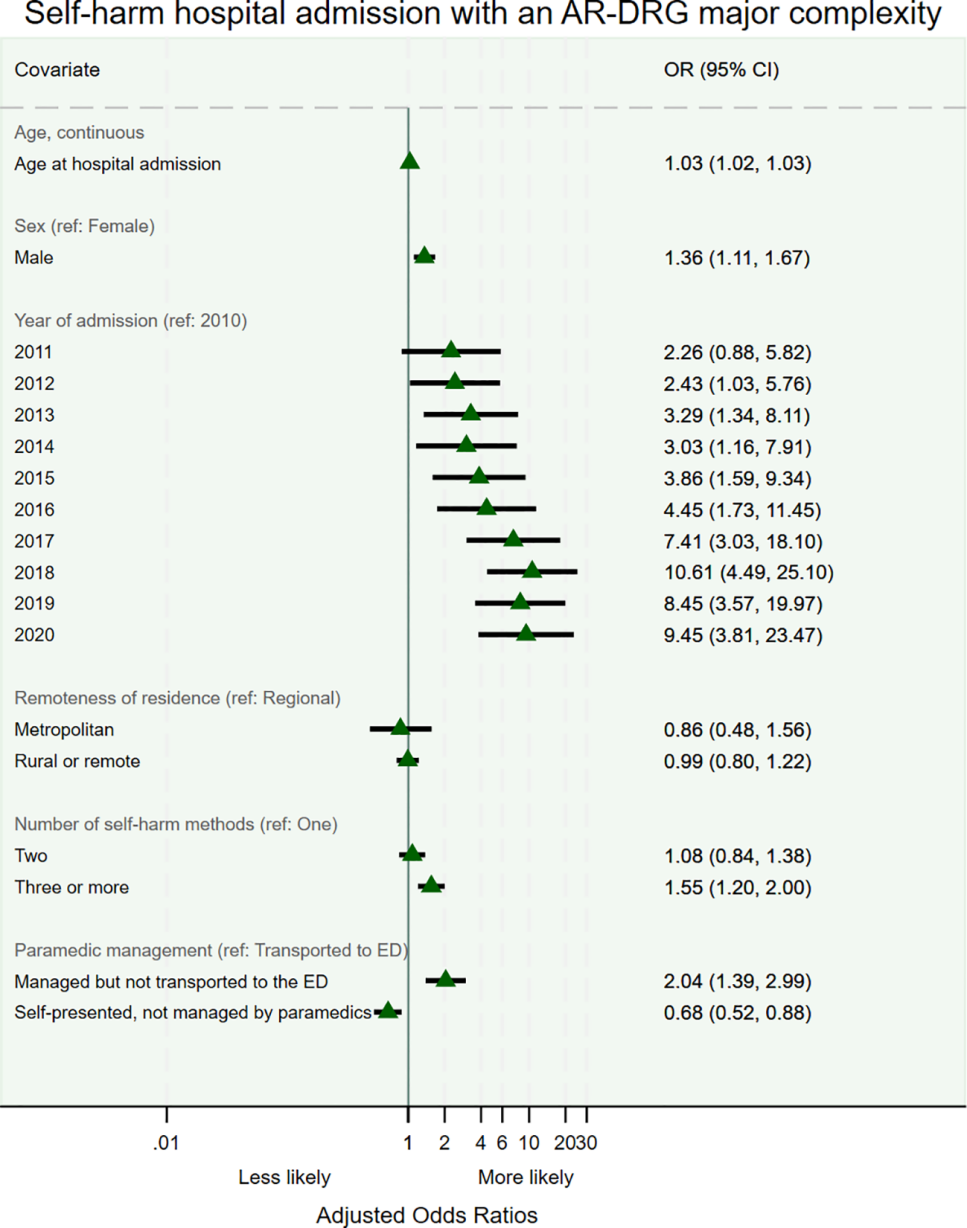
Odds of being admitted with a major AR-DRG complexity following self-harm: generalised estimating equations approach.

In the multivariable model that adjusted for demographics, year of admission, and number of self-harm methods used, patients assessed by paramedics but not transported to the ED were twice as likely to have a major AR-DRG complexity admission compared to those who were assessed and transported to the ED. The adjusted odds ratio (OR) was 2.04 (95% confidence interval (CI) 1.39–2.99), p<0.001 (**Figure 2**). The use of multiple self-harm methods independently increased the level of complexity, and males were also 36% more likely than females to be admitted with a major complex condition (OR 1.36 (95% CI 1.11 – 1.67).

No interactions were found between sex and year or between sex and AR-DRG complexity of presentation.

Sensitivity analysis that excluded patients whose self-harm intent was unknown supported the main study findings.

## Discussion

This population-based longitudinal study examined trends in the complexity of self-harm admissions over 11 years in a regional and rural context. Of the 2,000 patients admitted for self-harm, 58.6% were female, with a significant proportion from the two lowest socioeconomic status quintiles. Females were more likely to be admitted through the emergency department and have repeat self-harm admissions, while males had a higher rate of self-harm with unknown intent and were more likely to be admitted with major complexity. Self-harm methods varied by sex, with females more often using poisoning, and males more frequently using smoke, flames, and sharp or blunt objects. Over time, self-harm admissions became more complex, with an increased correlation between AR-DRG complexity and admissions to an intensive or coronary care unit.

This study highlights the growing complexity of self-harm admissions over time in both sexes, likely due to the increasing use of multiple self-harm methods, as shown in our findings. The use of multiple methods often leads to more complex injuries that require specialised medical care, contributing to higher complexity in hospital admissions (15). Other factors that may increase the complexity of self-harm presentations include the use of smoke, flames, and jumping from heights (16), although the increase in these methods over time was not observed in our study. The increased coding of complex conditions over time could also be attributed to improvements in medical reporting and classification, which could have enabled the identification and categorisation of more patients with severe conditions under higher complexity (17). However, alongside the rise in AR-DRG complexity, we also report a significant increase in ICU or CCU admissions, further supporting the trend of increasing complexity in self-harm cases. Additionally, the increase in complex cases may reflect a higher survival rate to hospital admission over time (18); however, this hypothesis cannot be proven in our study, as we did not capture all self-harm attempts in the community. Although the complexity of self-harm increased over time, similar to other reports (19), our study found no evidence to indicate an increase in self-harm admissions during the COVID-19 pandemic.

Self-harm and suicide are a complex reaction of multiple biological, sociological, environmental, and lifestyle risk factors that often interconnect to influence peoples’ risk of suicidal behaviours (20). However, suicide ideation, self-harm, and suicide are preventable (21). Literature consistently indicates that self-harm episodes are short-lived with the majority (70%) of individuals who had attempted suicide never attempting it again as shown in a systematic review that reviewed ninety follow-up studies (22). Of those who had attempted suicide, approximately 7% would die following a re-attempt with the remaining 23% non-fatally re-attempting suicide. This may indicate that risk of a repeat self-harm may wane as time passes although a past history of a suicide attempt is the strongest predictor of suicide (23).

Our study highlights the significant role that pre-hospital ambulance services have in managing self-harm. In our study, most of individuals admitted for self-harm sought help either by calling emergency services themselves or through others seeking help on their behalf. The extent to which help is usually sought when self-harming can vary depending on the method of self-harm, the individuals’ condition, impaired judgment, feelings of hopelessness, intention to self-harm, and other factors (24). In our study, we report that approximately 10% of those assessed by paramedics for self-harm within a 30-day period from the hospital admission, were not transported to hospital by the emergency services. It is unknown to us what proportion of those not transported to the ED refused transport as non-refusal factors can vary by age, sex, and condition (25, 26). Although transfer to hospital is not always the ideal management pathway of self-harm presentations in the prehospital setting (27, 28), our study indicates that the overall severity and complexity of self-harm presentations may be higher among patients managed by paramedics in the prehospital setting and not transported to hospital.

### Limitations

This study was limited to patients who self-harmed and survived to hospital admission; self-harm attempts in the community were not included. As the study focused on a single major regional hospital in Victoria, self-harm presentations in the broader community were not captured, and the findings may not be generalisable to other hospitals serving regional communities across Australia. Moreover, as it is likely that more complex cases were transferred to larger medical centres, our data are likely to have underestimated the true rates for the population. While we acknowledge that self-harm rates are higher among individuals who identify as Aboriginal or Torres Strait Islander (ATSI), we were unable to report results by ethnicity, as we had no information on patients’ ethnic backgrounds or their identification as ATSI. Lastly, since this study used routinely collected data, we lacked information on patients’ history of past self-harm attempts or suicidal ideation.

## Conclusion

This study provides evidence of increasing trends in complex self-harm admissions within a regional and rural population. Approximately 28% of the Australian population resides in regional and remote areas, and understanding the geographical distribution of self-harm admissions can help target self-harm prevention efforts in regions with the greatest need. Our findings underscore the importance of effective paramedic management of self-harm cases in the prehospital setting. Improving paramedic training to better assess and prioritise patients according to the severity of their condition is crucial (29). Furthermore, increasing access to mental health professionals in these settings could help identify patients in need of immediate psychiatric support.

## Data Availability

The original contributions presented in the study are included in the article/supplementary material. Further inquiries can be directed to the corresponding author/s.
